# Accelerometer-Based Automated Counting of Ten Exercises without Exercise-Specific Training or Tuning

**DOI:** 10.1155/2020/8869134

**Published:** 2020-10-10

**Authors:** Samuel Zelman, Michael Dow, Thasina Tabashum, Ting Xiao, Mark V. Albert

**Affiliations:** ^1^Illinois Math and Science Academy, Aurora, IL 60506, USA; ^2^Department of Computer Science and Engineering, University of North Texas, Denton, TX 76203, USA; ^3^Department of Biomedical Engineering, University of North Texas, Denton, TX 76203, USA; ^4^Department of Physical Medicine and Rehabilitation, Northwestern University Feinberg School of Medicine, Chicago, IL 60611, USA

## Abstract

Measuring physical activity using wearable sensors is essential for quantifying adherence to exercise regiments in clinical research and motivating individuals to continue exercising. An important aspect of wearable activity tracking is counting particular movements. One limitation of many previous models is the need to design the counting for a specific exercise. However, during physical therapy, some movements are unique to the patient and also valuable to track. To address this, we create an automatic repetition counting system that is flexible enough to measure multiple distinct and repeating movements during physical therapy without being trained on the specific motion. Accelerometers, using smartphones, were attached to the body or held by participants to track repetitive motions during different exercises. 18 participants completed a series of 10 exercises for 30 seconds, including arm circles, bicep curls, bridges, sit-ups, elbow extensions, leg lifts, lunges, push-ups, squats, and upper trunk rotations. To count the repetitions of each exercise, we apply three analysis techniques: (a) threshold crossing, (b) threshold crossing with a low-pass filter, and (c) Fourier transform. The results demonstrate that arm circles and push-ups can be tracked well, while less periodic and irregular motions such as upper trunk rotations are more difficult. Overall, threshold crossing with low-pass filtering achieves the best performance among these methods. We conclude that the proposed automatic counting system is capable of tracking exercise repetition without prior training and development for that activity.

## 1. Introduction

Physical therapy is a key strategy to improve mobility and quality of a patient's life after injuries, surgeries, and other debilitating events [1]. Physical therapy sessions take place in clinical settings with licensed physical therapists that help to instruct and encourage patients to increase comfort and mobility [2]. Unfortunately, these sessions may occur infrequently and therefore provide insufficient information about each patient's activities and efforts. Evaluation of physical therapy has always been considered as a challenge in research [3, 4]. An automated system for tracking exercises at home can play a vital role in improving patient's motivation and adherence to prescribed repetitive exercises as well as help to track and encourage patient's movements with minimum guidance from a physical therapist.

Wearable devices are the body-worn technologies that can perform computing functionalities during execution of physical tasks [5]. Such knowledge of users' physical status provides huge scope for utilizing wearable devices to assist clinicians to evaluate physical activity and can be applied to track posture in the clinical population [6]. Accelerometers are common sensors which are integrated in wearable technologies, and the use of accelerometers has escalated significantly over the past decade in the health-monitoring research area [7]. Using signal features extracted by improved modern accelerometers allowed experts to utilize those information in clinical science [8]. Despite the fact that the use of accelerometers has increased exponentially, reliable assessment of physical activity is still needed to explore [9]. However, it is also important to consider the technologies that are easily accessible and part of our daily life, such as cellphones. Most smartphones now incorporate a three-axis accelerometer sensor, and phone accelerometers are now used to perform activity recognition [10]. Implementing a simple step count can be achieved easily with the latest smartphones, but the activity tracking system without depending on labeled training data offers substantial research scope. There have already been some studies of activities on quantifying and recognizing using mobile phones [11–13], but this technology has not been fully examined for unknown activity.

In the context of physical therapy, the exercises depend on the specific conditions of every patient. Physical therapy exercises can differ significantly from one exercise to the next, which makes developing tailored recognition strategies challenging when the type of activities are unknown. One limitation with activity recognition models is that most of them are pretrained. People are instructed to make a particular movement (steps, walking, running, etc.), and data are acquired on that movement; then, offline analysis is performed to create a model to specifically measure that particular movement. Generally, machine learning techniques perform well on this kind of task when large volumes of data are available [14]. However, pretrained models require sufficient data for that particular movement to train and validate; this is difficult for unique instructed movements which can happen in physical therapy rehabilitation. Furthermore, periodic signals can offer a window of opportunities in various research problems. Many previous studies required large amounts of labeled data to achieve proper performance to count exercises. For example, to build RecoFit, a total 114 participants were recorded in over 146 sessions [15]. To address the concern of availability of quality data, we used the periodic signals and proposed methods that are adaptive. Our approach does not need labeled training data to perform automatic counting and it can be integrated with future work that aims to address real-time scenarios of repetition count.

In this study, we apply three straightforward analysis techniques to count the periodic motions. Ten different exercises were performed to assess not only the analysis strategies but also compare the quality of results for each exercise. Repeated exercises can help to perform automatic counting by using a repetitive structure of signals.

## 2. Materials and Methods

Smartphones with accelerometers have been placed on 18 subjects, aged 15–25 years, who completed a series of 10 different exercises for 30 seconds each, repeating each exercise twice, and we took the average of the two readings; however, differences in the count were rare. 7 females and 11 males participated in the study. These activities were arm circles, bicep curls, bridges, sit-ups, elbow extensions, leg lifts, lunges, push-ups, squats, and upper trunk rotations. We chose 10 common exercises that can be performed safely allowing untrained participants to execute the exercises properly. The exercises were also selected to involve a combination and coordination of muscles across the body. Three standard analysis techniques have been compared to count the number of repetitions of each activity: threshold crossing, threshold crossing with a low-pass filter, and a Fourier transform approach.

To collect data for this experiment, LG Optimus S smartphone accelerometers were used. The Sensor Log application (Sensor Log) recorded acceleration on 3 axes. The smartphone was either held by participants or strapped onto them, based on the exercise they were performing. The location of the smartphone for each exercise is listed in Table 1. Researchers found a similar level of accuracy for detecting everyday activities from a sensor placed in five different locations [16]. We decided to place the device according to what type of exercise, e.g., arm circle and elbow extension on hand and sit ups on the chest. The number of repetitions of each exercise was recorded by both the participant and the observer. Each exercise was repeated, and its reading was removed from analysis if the number of counted repetitions by participants and observers were not the same. Between each exercise, the participants were allowed an optional one-minute break. The smartphone's sensors were started at the beginning of each activity and were turned off at the end of the activity, to eliminate nonexercise data. The collected accelerometer data from smartphones were converted into magnitudes of acceleration and then trimmed manually only at the beginning and end of the 30-second session to obtain only the exercises and filter out nonactivity data. A sign multiplication was applied to the magnitude of the accelerometer in order to avoid a half-wave rectification of the signal. If the signal was in the direction of gravity (using a long-timescale moving average), the signal was positive and if opposite, it was negative. The experiments have been approved by the Illinois Math and Science Academy Institutional Review Board. It has been confirmed that all experiments were performed under relevant guidelines and regulations.

Three methods of estimating activity counts including threshold crossing, threshold crossing with a low-pass filter, and a Fourier transform approach have been used. Each method was written in Python, using the NumPy and SciPy + libraries. Each analysis technique is as follows.

### 2.1. Threshold Crossing

This technique has been utilized to calculate activity counts by tuning the threshold line position at two-third of the range between the minimum and maximum. When the accelerometer data cross the line in a positive direction, one count would be added. After adding each count, a refractory period of 0.1 seconds has occurred to prevent counting another repetition needlessly.

### 2.2. Threshold Crossing with Low-Pass Filtering

The technique was identical to the threshold crossing technique, except for the application of a Butterworth filter to minimize the impact of high-frequency noise. The low-pass filter method was performed using a moving average filter with the formula *A*_*i+1*_ *=* *0*.*9 A*_*i*_ + *0*.*1 X*_*i*_.

### 2.3. Fourier Transform

This method used a standard Fourier frequency decomposition, where the number of repetitions was calculated using the dominant frequency based on the length of time of the data collection. Count = frequency *∗* time.

The threshold level was the third quartile (30% percentile) of the magnitude distribution, which was chosen by maximizing accuracy using a grid sampling on a convenience sample for methods 2 and 3.

## 3. Results

**Figure 1:**
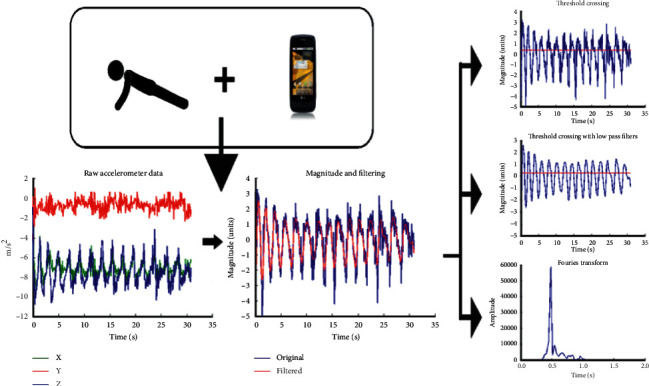
The data from the smartphone accelerometer is analyzed by applying three different counting methods. The 3-axis accelerometer reading is converted to a magnitude and processed further by (top) counting when the function crosses a threshold (middle) (threshold crossing) but after smoothing the magnitude (bottom) using the frequency indicated by a peak in the Fourier analysis to estimate count over time.

The general data analysis approach is depicted in Figure 1. Given this approach, it is expected that exercises with more periodic and large motions such as squats, push-ups, and arm circles will likely be counted with higher accuracy. The less periodic and smaller range of motion movements such as bridges, upper trunk rotations, and bicep circles are less accurately measured.  Table 2 records the accuracy of each method with the type of exercise performed by individuals. From the table, it can be seen that arm circles are counted well even by using the simple threshold method, likely due to a very small noise in the data and the periodic nature of the motion. On the other hand, upper trunk rotations were more difficult to count, especially with the simple threshold crossing method.

**Figure 2:**
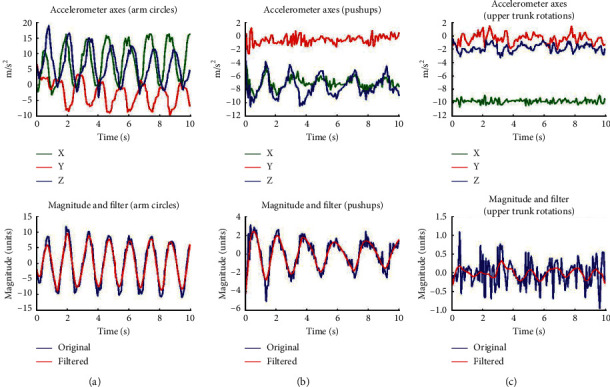
An observation of acceleration data for three different types of motions such as (a)arm circles (b) upper trunk rotation (c) bridges, representing more to less periodic motion, respectively.

Accelerometer data were recorded for 30 seconds along with manual counts of the exercises.  Figure 2 demonstrates the 3-axis accelerometer data and the acceleration magnitude for arm circles, push-ups, and upper trunk rotations noting the expected high-, medium-, and low-accuracy movements. We can observe in these magnitudes (Table 2) that exercises with more identifiable repetition in the signal and large motions such as squats, push-ups, and arm circles will likely be counted with higher accuracy. The less repetitive and smaller range of motion movements such as bridges, upper trunk rotations, and bicep circles may be less accurately measured.

In general, the results demonstrate that threshold crossing with a low-pass filter is the most effective counting method, with an average root mean square error of 8.69, noting a high variation depending on the movement being tracked. Fourier transform also performs reasonably well, with a root mean square error of 9.00. Threshold crossing without filtering does not perform well based on both of the roots mean square error of 13.38 as expected. Squats are the easiest exercise to count, whereas upper trunk rotations are the most difficult. Thus, we can conclude that periodic motions with substantial ranges are easiest to count.

## 4. Discussion

The goal of this study was to determine the accuracy of simple methods of counting repetitions from 10 different activities using an accelerometer. We applied three different methods of counting which include threshold crossing, threshold crossing with low-pass filtering, and the Fourier transform. We find that the best results for all counts belong to the large, periodic motion exercises, as expected.

To count motions accurately in practice, we need to address several different challenges. Since accelerometer data varied depending on the exercise being studied, we did not tailor the counting method to each specific activity. The chosen counting methods are flexible and can be used on any patient, even those with abnormal movement patterns. This flexible approach has an additional benefit of making these methods more robust; the accuracy achieved without utilizing automatic segmentation or recognition. It can count activities without having prior knowledge of the type of activity. Notably, there are limitations to this overall strategy. We are unable to count more than one distinct activity taking place in the same period, which can be particularly problematic when accounting for prolonged moments of rest between motions. Another constraint is that the experiment was conducted in a limited age group (between 15 and 25). For older people, it might be different and more challenging. In this case, the duration of the reading for each exercise should be considered as it will take more time to complete each repetition and there might be more noise in accelerometer data. Further critical decision is the location of an accelerometer.

Machine learning-based activity recognition models can provide accurate counts when the particular activity being counted is pretrained. There are numerous studies to show the reliability and accuracy of accelerometer-based activity measurements [17]. Fall detection relies on accurate training data [18, 19]. ReadySteady, an accelerometer-based mobile application for activity assessment, has been developed, which shows that the system can differentiate the intensity of any activity, for example, sitting, driving, and walking [20]. A previous study analyzed the repetition accuracy of weight training and calisthenics through an arm-worn accelerometer [15]. Additionally, activity recognition can be improved through the use of dynamic state estimators such as hidden Markov models [21, 22]. The use of segmentation and activity recognition allowed the researchers to improve repetition count accuracy, as well as identifying exercise data from nonexercise data. They also discussed how individual learning models can improve counting accuracy for the activities, once activities are segmented and classified.

Further studies demonstrated using a routine tracking of health can motivate and encourage positive health behaviors [23, 24]. For instance, researchers at Stanford found that pedometer users had a 26.9% increase in activity over baseline [25]. Cadmus–Bertham illustrates that supplying Fitbits to postmenopausal women led to increased physical activity, over a period of 16 weeks [26]. Having a simple way to collect detailed exercise data at home can help physical therapists tailor their sessions in the clinic, which provides a large benefit to physical care. In summary, we evaluated the effectiveness of three different analysis methods in the counting of exercises. The error rate is higher than it is expected to be considered usable, primarily as there was no tuning to individual activities. The location of the accelerometer is one concern. It is important to note that without any prior knowledge of the data, we can see a pattern of how repetitive exercises can be tracked using our method. The most effective method for each task varied, due to different amounts of noise and different levels of periodicity. Threshold crossing works well with arm circles and crunches. After adding a smoothing filter to remove noise and high-frequency motion threshold crossing also works well for lunges, pushups, and squats, with higher general performance in all activities other than arm circles and crunches. The Fourier transform approach works exceptionally well for lunges and squats but generally performs poorly overall. These methods can serve as effective baselines for unspecified periodic activity counting.

In the future, we plan to perform and test our method on clinical populations including older subjects and individuals with disabilities. In addition, machine learning methods can be explored; this is an extensive approach used in human activity recognition, but commonly, it is applied to create pretrained models for specific movements. Variations of training paradigms will be explored to use machine learning without identifying the type of movement.

## 5. Conclusion

In this study, we performed three analysis techniques to achieve automatic counting of unseen exercises. The repetitive motions of ten exercises of eighteen participants were evaluated through (a) threshold crossing, (b) threshold crossing with a low-pass filter, and (c) Fourier transform. From the result, we can conclude that less periodic activities are more prone to error, whereas the periodic motions can be tracked easily using our approach. This study can be applied to perform and evaluate exercises at home with minimal help from a physical therapist. Therefore, automatic counting methods help patients to save time and money by decreasing the number of rehabilitation sessions and encouraging therapy compliance. This study would have an extended effect on physical therapy and, ultimately, such tracking can provide clinical benefit to patients and therapists.

## Figures and Tables

**Table 1:** Location where the smartphone is placed and how it is held by the individual during the exercise.

Exercise	Location	Holding methods
Arm circle	Hand	Held in hand: random orientation
Bicep curl	Hand	Held in hand: random orientation
Bridge	Waist	Pouch
Crunch	Chest	Held in hands with hands on positioned on chest
Elbow extension	Hand	Held in hand: random orientation
Lower trunk rotation	Waist	Pouch
Lunge	Waist	Pouch
Push ups	Waist	Pouch
Squats	Waist	Pouch
Upper trunk rotation	Waist	Pouch

**Table 2:** Root mean square error (RMSE) of counts for each analysis technique.

Exercise	Threshold crossing	Threshold with low pass	Fourier	Avg RMSE
Arm circle	1.99	9.65	8.08	7.36
Bicep curl	16.70	7.36	12.29	12.71
Bridge	13.94	14.39	7.07	12.27
Crunch	7.32	8.56	12.18	9.58
Elbow extension	13.70	10.20	10.57	11.59
Lower trunk rotation	13.47	9.97	7.45	10.59
Lunge	16.95	1.83	0.76	9.85
Push-ups	10.53	1.39	3.70	6.49
Squats	10.71	1.50	0.93	6.27
Upper trunk rotation	19.06	9.77	14.11	14.81

Avg RMSE	13.38	8.69	9.00	

Threshold crossing with a low-pass filter performs best with an average root mean square error of 8.69, with the Fourier transform being close with 9.00.

## Data Availability

The data used to support the findings of this study have not been made available.
